# Imaging structural and functional brain networks in temporal lobe epilepsy

**DOI:** 10.3389/fnhum.2013.00624

**Published:** 2013-10-01

**Authors:** Boris C. Bernhardt, SeokJun Hong, Andrea Bernasconi, Neda Bernasconi

**Affiliations:** ^1^Neuroimaging of Epilepsy Laboratory, Montreal Neurological Institute and Hospital, McGill UniversityMontreal, QC, Canada; ^2^Department of Social Neuroscience, Max Planck Institute for Human Cognitive and Brain SciencesLeipzig, Germany

**Keywords:** TLE, connectivity, MRI, graph-theory, connectome

## Abstract

Early imaging studies in temporal lobe epilepsy (TLE) focused on the search for mesial temporal sclerosis, as its surgical removal results in clinically meaningful improvement in about 70% of patients. Nevertheless, a considerable subgroup of patients continues to suffer from post-operative seizures. Although the reasons for surgical failure are not fully understood, electrophysiological and imaging data suggest that anomalies extending beyond the temporal lobe may have negative impact on outcome. This hypothesis has revived the concept of human epilepsy as a disorder of distributed brain networks. Recent methodological advances in non-invasive neuroimaging have led to quantify structural and functional networks *in vivo*. While structural networks can be inferred from diffusion MRI tractography and inter-regional covariance patterns of structural measures such as cortical thickness, functional connectivity is generally computed based on statistical dependencies of neurophysiological time-series, measured through functional MRI or electroencephalographic techniques. This review considers the application of advanced analytical methods in structural and functional connectivity analyses in TLE. We will specifically highlight findings from graph-theoretical analysis that allow assessing the topological organization of brain networks. These studies have provided compelling evidence that TLE is a system disorder with profound alterations in local and distributed networks. In addition, there is emerging evidence for the utility of network properties as clinical diagnostic markers. Nowadays, a network perspective is considered to be essential to the understanding of the development, progression, and management of epilepsy.

## Introduction

Epilepsy is one of the most prevalent neurological disorders, affecting ~1% of the general population. Of patients treated with antiepileptic drugs, about one third never achieve remission. Drug-resistance should be identified early and treated effectively, as uncontrolled epilepsy is harmful to the brain, has devastating socio-economic consequences, and is associated with increased mortality (Leonardi and Ustun, 2002; Pugliatti et al., 2007; Tellez-Zenteno et al., 2007; Cascino, 2009; Coan and Cendes, 2013). Epilepsy is broadly defined by a state of recurrent spontaneous seizures, which arise when balance between excitation and inhibition is disrupted (Scharfman, 2007; Engel et al., 2013). Compelling evidence from animal models, experimental paradigms, and clinical work in humans indicates that specific cortical and subcortical networks play a fundamental role in the genesis and expression of seizures (Avoli and Gloor, 1982; Bear et al., 1996; Bertram, 1997; Spencer, 2002).

Temporal lobe epilepsy (TLE) is the most common drug-resistant epilepsy in adults. TLE is traditionally associated with mesiotemporal sclerosis, defined by cell loss and gliosis in the hippocampus, entorhinal cortex, and amygdala. Surgical resection of this epileptogenic lesion is the current treatment of choice (Wiebe et al., 2001), and leads to freedom from seizures in the majority of cases. Nevertheless, even in carefully selected cases, ~30% of surgical candidates continue to have seizures (Mcintosh et al., 2004; Bernhardt et al., 2010). Although reasons for surgical failure are not fully understood, electrophysiological and imaging data suggest that anomalies extending beyond the temporal lobe may have negative impact on outcome. This hypothesis has revived the concept of human epilepsy as a disorder of distributed neural networks (Spencer, 2002; Bonilha et al., 2007b; Elsharkawy et al., 2009; Engel et al., 2013).

In the past decade, advances in imaging acquisition and postprocessing have permitted *in vivo* mapping of the regional distribution of network abnormalities in TLE patients. In particular, quantitative structural MRI studies based on volumetry, voxel-based morphometry, cortical thickness mapping, and structural covariance analysis have shown widespread, coordinated, and progressive cortical gray matter loss in temporal and extra-temporal regions, such as the thalamus, fronto-limbic, and fronto-central neocortices (Bernasconi et al., 2003b, 2004; Natsume et al., 2003; Bonilha et al., 2004; Lin et al., 2007; Bernhardt et al., 2008, 2009, 2010, 2012; Keller and Roberts, 2008; Mcdonald et al., 2008b,c). Findings of gray matter alterations have been complemented by diffusion MRI data of the white matter. These studies have shown disruptions in inter-regional fiber diffusivity both within and beyond mesiotemporal and temporo-limbic networks, suggestive of decreased fiber arrangement and altered myelin membranes (Concha et al., 2005; Rodrigo et al., 2007; Yogarajah and Duncan, 2008; Ahmadi et al., 2009). Furthermore, studies based on both electrophysiological techniques as well as functional MRI have provided evidence for region-specific shifts in intrinsic functional networks (Bettus et al., 2009; Voets et al., 2012). More recently, reports of disruptions of inter-regional structural and functional connections in TLE have been complemented by graph-theoretical approaches (Liao et al., 2010; Bernhardt et al., 2011; Bonilha et al., 2012). These techniques, derived from complex system analysis, lend tools to characterize topological aspects that relate to the specialization and integration of inter-connected brain networks (Watts and Strogatz, 1998; Sporns et al., 2004; Bullmore and Sporns, 2009; Guye et al., 2010). In TLE, such approaches provide a novel window to study connectivity, and have begun showing alterations in higher-order network configurations.

The aim of this review is to summarize the current state of imaging evidence for network abnormalities in TLE. We will first outline findings that have provided insights into the topographical extent of regional structural abnormalities in TLE. We will then discuss studies on low-level inter-regional abnormalities, using connectivity mapping techniques such as seed-based structural MRI covariance, functional MRI connectivity, and diffusion MRI tractography. Subsequently, we will discuss graph-theoretical analyses to address the topological organization of brain networks in TLE. We will conclude by commenting on the potential clinical relevance of current network-based MRI analysis in TLE.

## Regional patterns of structural pathology

The hallmark lesion of TLE is hippocampal sclerosis. This lesion is characterized by various degrees of neuronal loss and gliosis within hippocampal subfields and the dentate gyrus (Sommer, 1880; Babb and Brown, 1987; Blumcke et al., 2002). In addition, up to 50% of TLE patients may show intense reorganization of neuronal networks, manifested by granule cell dispersion (Houser et al., 1992; Blumcke et al., 2002), selective loss of inhibitory neurons (De Lanerolle et al., 1989), as well as axonal sprouting (Babb et al., 1991). Histological reports of TLE patients and animal models of limbic epilepsy have consistently demonstrated that pathology is not limited to the hippocampus. Indeed, cell loss and gliosis may be found in proximal and even more distal temporo-limbic regions, including the amygdala (Yilmazer-Hanke et al., 2000), entorhinal cortex (Du et al., 1993, 1995), temporopolar (Choi et al., 1999; Meiners et al., 1999; Mitchell et al., 1999; Bothwell et al., 2001) and lateral temporal neocortices (Cavanagh and Meyer, 1956; Falconer et al., 1964; Turski et al., 1983; Clifford et al., 1987; Kuzniecky et al., 1987; Cavalheiro et al., 1991; Thom et al., 2009), as well as the thalamus (Turski et al., 1983; Clifford et al., 1987; Bertram et al., 2001; Sloan and Bertram, 2009). In animal models, tissue damage has been shown in extra-temporal neocortical regions, such as sensorimotor cortex, piriform, perirhinal, retrosplenial, and visual cortices. In humans, pathological data in regions remote from the temporal lobes in TLE is sparse. This is, in part, due to difficulties in obtaining immediate *postmortem* specimens and the surgical approach tailored to the temporal lobe. In their seminal postmortem study, Margerison and Corsellis described neuronal loss and gliosis in frontal and occipital cortices in about 20% of patients (Margerison and Corsellis, 1966). More recent autopsy reports have confirmed and further extended these observations by showing varying degrees of architectural abnormalities involving virtually all lobes (Eriksson et al., 2002; Blanc et al., 2011).

**Figure 1:**
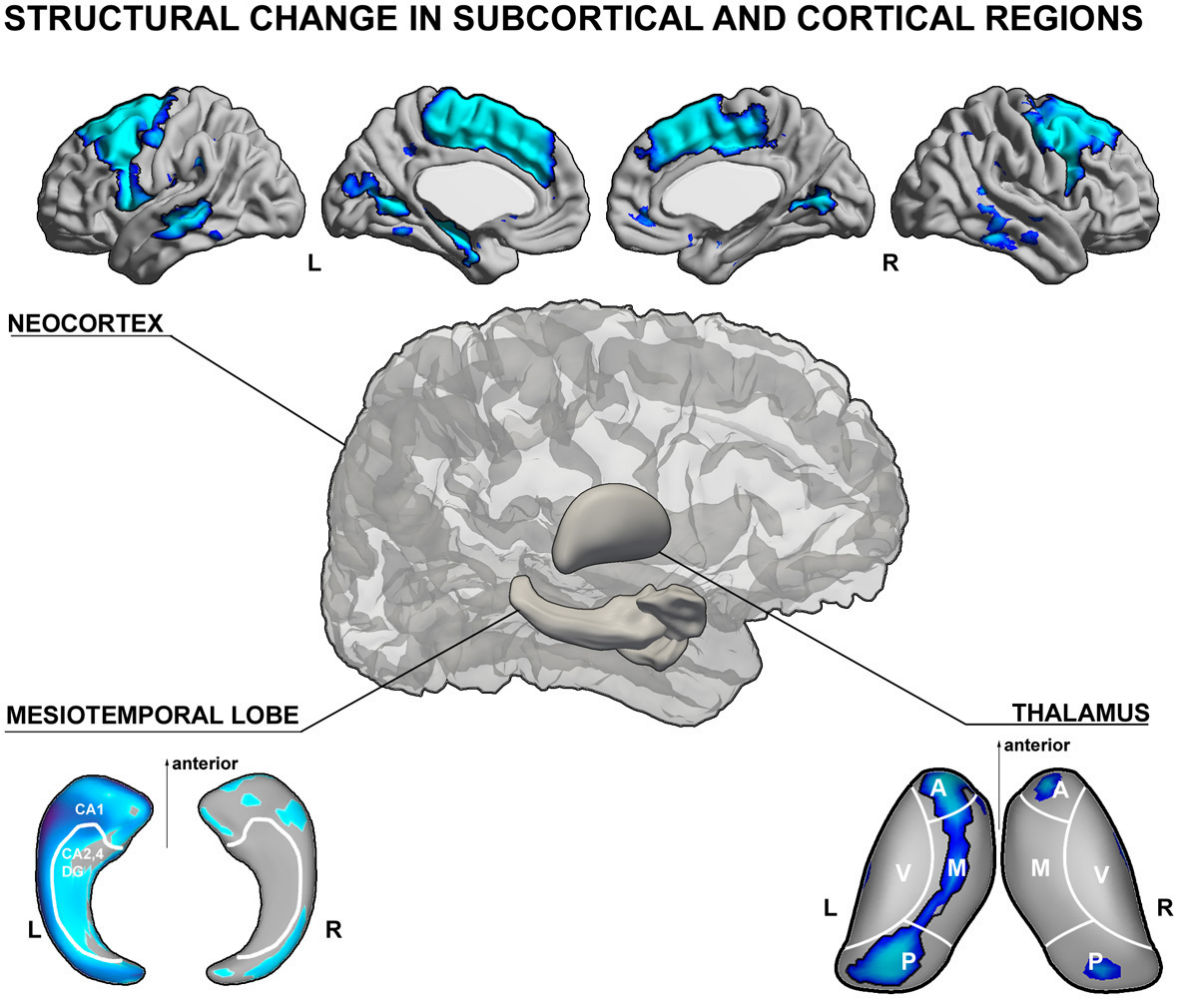
**Schematic illustration of gray matter structural anomalies in temporal lobe epilepsy. (top)** Results from MRI-based cortical thickness analysis, showing cortical thinning in left temporal lobe epilepsy (TLE) patients with hippocampal atrophy relative to healthy controls in mesial and lateral temporal as well as fronto-central neocortices (Bernhardt et al., 2010). **(lower left)** Patterns of atrophy in TLE patients relative to controls in CA1 subregions of the ipsilateral hippocampus (Kim et al., 2008) and **(lower right)** mediodorsal segments of the thalamus (Bernhardt et al., 2012), both generated using spherical harmonic surface-shape modeling techniques of manual MRI segmentations. The shown analyses have been generated using the SurfStat toolbox for Matlab (Worsley et al., 2009). Further details on the statistical procedures can be found in the original publications.

A large body of electro-clinical work suggests that the epileptogenic network in TLE is broad. Seizure activity may involve not only the hippocampus, but also several other subcortical and cortical structures, including the amygdala, entorhinal cortex, lateral temporal, inferior, as well as orbitofrontal cortices (Lieb et al., 1987, 1991) together with the medial thalamus (Cassidy and Gale, 1998; Rosenberg et al., 2006). The close spatial correspondence between histopathological alterations and electrophysiological anomalies in TLE has provided a strong motivation to study structural brain changes, which have been of a high clinical and scientific value in mapping causes and consequences of drug-resistant epilepsy. In particular, quantitative Magnetic Resonance Imaging (MRI) analysis has offered a unique perspective to study structural substrates of TLE *in vivo* and to gain further insights into their spatial patterns and clinical correlates (See Figure 1, for a schematic overview of structural MRI findings in TLE). Studies based on manual volumetric MRI analysis largely confirmed previous histological assessments, and provided a more comprehensive picture of the regional extent of structural abnormalities in TLE. Volumetric analysis demonstrated atrophy in multiple limbic structures, including the hippocampus, entorhinal cortex, amygdala (Cendes et al., 1993a,b; Bernasconi et al., 2001, 2003a,b), temporopolar, perirhinal, lateral temporal neocortices (Jutila et al., 2001; Moran et al., 2001; Sankar et al., 2008), and the thalamus (Dreifuss et al., 2001; Natsume et al., 2003; Bernhardt et al., 2012). In the hippocampus and thalamus, surface shape mapping has furthermore allowed localizing structural anomalies at a subregional level (Hogan et al., 2004; Kim et al., 2008; Bernhardt et al., 2012, in press). In the thalamus, for example, we found volume loss located primarily in mediodorsal segments (Bernhardt et al., 2012). Quantitative MRI postprocessing techniques, such as voxel-based morphometry (Bernasconi et al., 2004; Bonilha et al., 2004; Keller and Roberts, 2008) and analyses of cortical thickness have shown that TLE is associated with extensive regional neocortical abnormalities, encompassing not only mesiotemporal structures, but also prefrontal, fronto-central, cingulate, occipito-temporal, and lateral temporal neocortices (Lin et al., 2007; Bernhardt et al., 2008, 2009, 2010, 2012; Mcdonald et al., 2008c; Mueller et al., 2009b; Kemmotsu et al., 2011; Voets et al., 2011). Although the exact biological underpinnings of gray matter loss in different brain regions are not clear, they likely reflect a combination of neuronal loss and synaptic reorganization (Cascino et al., 1991; Sanabria et al., 2002; Blanc et al., 2011), possibly secondary to seizures (Sutula et al., 1988; Holmes, 2002; Cavazos et al., 2003). These findings have increased our understanding of whole-brain pathology associated with TLE. On the other hand, new techniques such as hippocampal and thalamic surface-shape mapping (Kim et al., 2008; Bernhardt et al., 2012) have allowed searching for fine-grained, subregional structural anomalies within temporo-limbic seizure networks. Importantly, the MRI-derived knowledge of pathology is in overall agreement with data from animal models and *ex vivo* studies. These studies collectively support the concept of TLE as a disorder of distributed neural networks.

## Structural networks

Quantitative structural studies have provided a comprehensive mapping of structural pathology in TLE. Nevertheless, the commonly applied mass-univariate group comparisons provide only a snapshot of putative network abnormalities in TLE. Indeed, while such topographic maps may localize an ensemble of affected regions, they do not directly address how these regions inter-relate.

The term *structural connectivity* refers to anatomical associations between brain regions, defining the actual physical wiring (Stephan et al., 2000; Stone and Kotter, 2002; Sporns et al., 2005; Sporns, 2011). The gold standard to define such connections has been anterograde and retrograde tract-tracing techniques. Tracers show good accuracy and sensitivity, in particular for mapping long-range connections, and have resulted in a rich and detailed cartography of connectivity in several mammalian species (Felleman and Van Essen, 1991; Scannell et al., 1995; Modha and Singh, 2010). Their invasiveness however, limits their application to animal studies (Sporns, 2011).

**Figure 2:**
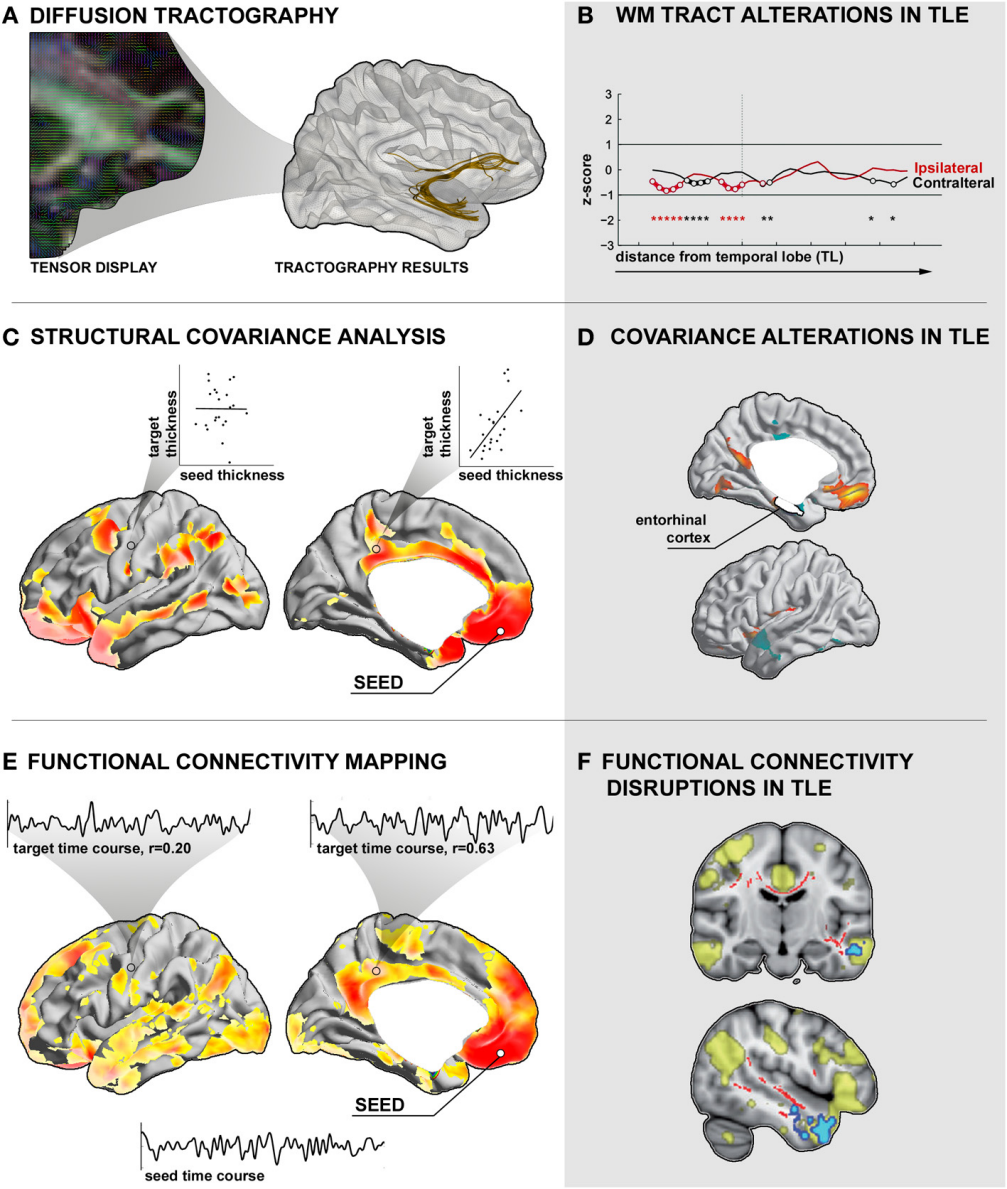
**Assessment of inter-regional connectivity. (A)** Diffusion tractography. *Left:* Illustration of diffusion tensor directions superimposed on a fractional anisotropy map derived from diffusion MRI. *Right*: Seed-based deterministic tractography of the uncinate fasciculus. **(B)** Altered mean diffusivity along the uncinate fasciculus tract in a group of patients with temporal lobe epilepsy (TLE) relative to controls (Concha et al., 2012). Prior to analysis, the tract was subdivided into bins with respect to the anatomical distance to the temporal and frontal lobes. **(C)** Structural covariance analysis. Shown is the cortical thickness correlation map of the left medial orbital cortex seed with the remaining cortical mantle in a group of healthy controls. High positive correlations are interpreted as connections, low correlations as absence of connections. **(D)** Structural covariance alterations in TLE patients relative to controls between an entorhinal cortex seed and target regions in medial orbitofrontal cortices (Bernhardt et al., 2008). **(E)** Functional connectivity between a left medial orbitofrontal cortex seed and the rest of the cortical mantle in healthy controls. Insets show exemplary time courses of the seed region with selected cortical target regions with high and low correlations, respectively. **(F)** Voxel-wise functional connectivity abnormalities in TLE, highlighting target regions with altered time-series correlation to a spatial component that closely matches the “default mode” network (Voets et al., 2012).

In humans, two major indirect approaches have been employed to map structural networks: *diffusion MRI tractography* and *structural MRI covariance* (see Figures 2A–D). Structural networks derived from diffusion-weighted MRI data provide an approximation of the underlying white matter architecture (Le Bihan et al., 1986, 1996; Johansen-Berg and Behrens, 2006; Jbabdi and Johansen-Berg, 2011) by describing the directionality and magnitude of water diffusion at each imaging voxel. These data can be further processed by tractography algorithms (Mori et al., 1999; Behrens et al., 2003), which reconstruct fiber pathways running along plausible diffusion trajectories in voxel-space (Figure 2A). While somewhat challenged in regions where different fiber populations intersect (Behrens et al., 2003; Jones et al., 2012), such as the cortical gray matter, tractography can generate consistent results, particularly in deep white matter. Findings have shown overall a good correspondence with the animal tracing literature, and have been cross-validated by comparative sacrificial tracing studies in non-human primates (Mori et al., 1999; Parker et al., 2002; Dauguet et al., 2007). Moreover, it has been shown that factors such as fiber diameter and density, membrane permeability, myelination, as well as fiber packing (Beaulieu, 2002; Concha et al., 2010) can influence the directionality and magnitude of water displacement at a given voxel. Diffusion imaging may, thus, be used to assess microstructural and architectural integrity *in vivo*. The most widely used diffusion tensor parameters are fractional anisotropy (FA), an index of deviation of water diffusion from a random spherical displacement, and mean diffusion (MD), a scalar marker of bulk diffusion at each voxel.

In TLE, previous diffusion MRI studies have consistently shown decreased FA in temporo-limbic tracts such as the fornix pathway (Concha et al., 2005; Ahmadi et al., 2009), parahippocampal fibers (Mcdonald et al., 2008a; Yogarajah and Duncan, 2008; Ahmadi et al., 2009), the uncinate fasciculus (Rodrigo et al., 2007; Diehl et al., 2008; Lin et al., 2008; Mcdonald et al., 2008a), and the cingulum bundle (Concha et al., 2008; Ahmadi et al., 2009), as well as in several frontal and posterior fiber tracts including the inferior and superior longitudinal fascicles (Focke et al., 2008; Lin et al., 2008; Mcdonald et al., 2008a; Ahmadi et al., 2009), the internal and external capsule (Arfanakis et al., 2002; Gross et al., 2006; Concha et al., 2008), and the corpus callosum (Arfanakis et al., 2002; Gross et al., 2006; Concha et al., 2008). Relative to the widespread pattern of FA changes, MD anomalies follow a more restricted distribution (Concha et al., 2005, 2008; Focke et al., 2008). In a recent study that assessed diffusion abnormalities along fiber tracts, our group could show that the effect size of MD alterations in TLE seems to decrease as a function of anatomical distance to the temporal lobe (Figure 2B), suggesting co-localization of these changes with the seizure focus (Concha et al., 2012).

The combined contribution of different microstructural and architectural properties to the diffusion signal precludes a straightforward, and universal biological interpretation of diffusion tensor indices and their alteration in disease (Jones et al., 2012). Diffusion MRI is, nevertheless, currently the only imaging method that can assess fiber architecture *in vivo* (Jones et al., 2012). Initial evidence from histopathological analysis of the fimbria-fornix pathways in operated TLE patients suggests that FA decreases may primarily reflect alterations in axonal membranes (Concha et al., 2010). MD changes, on the other hand, have been shown to vary with respect to the dynamics of seizure activity (Yu and Tan, 2008; Concha et al., 2012). Indeed, MD has been shown to decrease in the hyperacute phase after prolonged seizures or status epilepticus, likely due to intracellular cytotoxic edema. Conversely, few days following the subacute peri-ictal phase, MD may increase as a consequence of vasogenic edema (Scott et al., 2006). Neuronal loss and gliosis can lead to further MD increase as a consequence of the chronic expansion of the interstitial water content.

Structural networks may also be studied through covariance analysis of MRI-based morphological metrics, such as cortical thickness or gray matter volume (Bullmore et al., 1998; Mechelli et al., 2005; Lerch et al., 2006; Bernhardt et al., 2008, 2013). According to the framework of MRI covariance analysis, a high correlation in morphological markers between two regions across subjects can be interpreted as a network link, while a low correlation indicates no link (Figure 2C). Similar to diffusion tractography, this correlational framework does not infer direct anatomical connections between pairs of regions. Nonetheless, analyzing structural covariance may detect manifestations of persistent functional-trophic cross-talk, maturational inter-change, as well as common developmental and pathological influences (Lerch et al., 2006; Bullmore and Sporns, 2009; Zielinski et al., 2010; Bernhardt et al., 2011; Raznahan et al., 2011; Xia and He, 2011; Khundrakpam et al., 2012; Alexander-Bloch et al., 2013). One of the advantages of cortical thickness covariance analysis is the direct seeding from cortical gray matter regions in a high-resolution space that is in principle not limited by the imaging voxels of the underlying MR image, but by the sampling density of the points on the cortical mesh. Correlation analysis of structural features may furthermore represent a relatively pragmatic approach toward structural network mapping. In fact, the commonly used T1-weighted images, a standard component of every clinical imaging protocol, have a short acquisition time. Moreover, these images are generally unaffected by distortion and signal dropout artifacts in orbitofrontal and temporo-basal regions often occurring in echo-planar functional and diffusion MRI sequences.

In TLE, several recent covariance analyses have mapped abnormal structural correlations between mesiotemporal and neocortical regions (Bonilha et al., 2007a; Bernhardt et al., 2008; Mueller et al., 2009b), thalamic and neocortical regions (Mueller et al., 2009a; Bernhardt et al., 2012), and within cortico-cortical networks (Mueller et al., 2009a). Correlating the thickness of the entorhinal cortex to that of the neocortex, our group found decreased structural coordination between mesial temporal regions and lateral temporal neocortices, suggestive of a connectional breakdown within temporo-limbic circuits (Figure 2D; Bernhardt et al., 2008). Moreover, covariance analysis of thalamo-cortical circuits (Hetherington et al., 2007; Mueller et al., 2009a; Bernhardt et al., 2012) has shown coupled structural and metabolic change of the thalamus with neocortical (Bernhardt et al., 2012) and with mesiotemporal regions (Hetherington et al., 2007; Mueller et al., 2009a), emphasizing a key role of this structure in the pathological network of TLE.

Diffusion MRI and structural MRI covariance analysis tap into different facets of structural brain networks. While diffusion MRI analysis may be the method of choice to study white matter tracts, and their potential architectural disruptions, structural covariance analysis may sensitively assess alterations in the trophic-morphological coordination between gray matter regions. Both approaches have advanced our understanding of the fundamental architecture of inter-regional connections, and their disruptions in TLE.

## Functional networks

The study of functional networks helps to elucidate *how* a structural architecture gives rise to alterations in neurophysiological dynamics. The term *functional connectivity* refers to the strength of statistical dependencies of neurophysiological signals between regions (Figure 2E).

Functional connectivity can be determined from time-series measured by functional MRI (Friston et al., 1993, 1996; Focke et al., 2008; Smith, 2012) or electrophysiological techniques, such as electroencephalography (EEG) (Lopes Da Silva et al., 1989; Tononi et al., 1994; Lachaux et al., 1999). Although functional MRI and EEG have complimentary temporal/spatial resolution tradeoffs, they can also be combined (Lemieux et al., 2011). In short, functional MRI does not directly measure neural activity, but only activity-dependent hemodynamic alterations, and has a relatively low temporal resolution in the range of 1–2 s [but see Feinberg et al. (2010); Smith et al. (2012), for a recent example of increasing the temporal resolution in functional MRI acquisitions]. Yet, this technique offers high spatial resolution in the millimeter range and allows imaging the entire brain (Lemieux et al., 2011). EEG, on the other hand, has a superior temporal resolution (in the order of milliseconds) but suffers from neurophysiological signals limited to the scalp.

One way to assess functional connectivity between different brain regions is through analysis of task-free (or, resting-state) paradigms, functional acquisitions during which the subject does not perform any task (Biswal et al., 1995, 2010; Greicius et al., 2003; Smith et al., 2009). Functional connectivity analysis of such task-free datasets has allowed the identification of brain networks that show strong coupling of intrinsic, spontaneous brain activity. Ample recent resting-state functional MRI assessments have revealed networks which are generally reproducible across subjects (Damoiseaux et al., 2006) that closely correspond to brain systems engaging in specific tasks (Biswal et al., 1995; Smith et al., 2009; Laird et al., 2011). Several studies have furthermore begun to explore the relationship between low-frequency resting-state networks derived from functional MRI and those measured from EEG (De Pasquale et al., 2010; Jann et al., 2010; Musso et al., 2010; Yuan et al., 2012). Moreover, several studies in primates have suggested a close correspondence between intrinsic functional MRI connections and known anatomical pathways (Mantini et al., 2011; Shen et al., 2012). In turn, other studies have demonstrated the utility of resting-state patterns to generate regional parcellations of specific anatomical areas (Margulies et al., 2007; Mars et al., 2011). Finally, analysis of resting-state connectivity patterns may be sensitive to detect disruptions of brain organization in disease conditions (Greicius, 2008; Fox and Greicius, 2010; Kelly et al., 2012).

Several EEG and combined EEG-fMRI studies have shown dynamic alterations in functional activations and connectivity patterns related to epileptic spikes (Gotman et al., 2006; Kobayashi et al., 2006; Laufs et al., 2007; Ponten et al., 2007; Bettus et al., 2011). Resting-state functional EEG and functional MRI connectivity analyses in TLE have also quantified chronic, inter-ictal changes in functional networks (Waites et al., 2006; Bettus et al., 2009). These studies have mainly focused on assessing associations of intrinsic signals between regions known to be involved in seizure activity, particularly among medial temporal lobe structures. Bettus and colleagues reported decreased functional connectivity in mesiotemporal regions proximal to the seizure focus; interestingly, ipsilateral decreases co-occurred with increased functional connectivity in contralateral regions (Bettus et al., 2009). Findings of contralateral connectivity increases are suggestive of compensatory network reorganization. Several studies have also suggested functional connectivity alterations in regions that comprise the “default mode” network (Raichle et al., 2001; Greicius et al., 2003; Voets et al., 2012), or between this network and other brain regions (Frings et al., 2009; Liao et al., 2010; Zhang et al., 2010). The default mode network includes a collection of medial frontal, midline parietal, and lateral parietal regions that show increased activation in the absence of a specific tasks, and whose function may closely relate to internal thought processes such as memory and mind-wandering (Buckner and Carroll, 2007; Christoff et al., 2009).

Changes in inter-regional functional coupling are thought to represent compensatory mechanisms secondary to structural pathology and seizure-related activity. Combining structural and functional image analysis, for example, our group recently showed disruptions in functional connectivity between mesiotemporal regions and neocortical target networks (Figure 2F) that may relate to abnormal gray matter density and altered diffusivity of inter-connecting fiber tracts (Voets et al., 2012) suggestive of a complex derangement in the structural-functional cross-links. There is also evidence for abnormal signal interactions in epileptic patients without a visible lesion on MRI (Vlooswijk et al., 2011). In addition, functional abnormalities have even been shown in regions unaffected by epileptic discharges (Bettus et al., 2011), suggestive of a widespread pathological process that alter the whole-brain intrinsic functional network architecture in TLE.

## Graph theory—modeling network topology

Conventional analysis approaches, mainly based on between-group comparisons have shown low-level regional and connectional alterations in TLE. These methods, however, are not tailored at capturing the complexity of whole-brain pathological interactions in TLE, which may affect higher-order, topological aspects of brain network organization.

Graph theory is a framework for the mathematical representation and analysis of complex systems. It has been applied to the analysis of artificial and biological networks (Watts and Strogatz, 1998). Graph theoretical analysis has recently attracted considerable attention in brain research because it provides a powerful formalism to quantitatively describe the topological organization of connectivity (Bullmore and Sporns, 2009; Guye et al., 2010; Bassett and Gazzaniga, 2011; Bullmore and Bassett, 2011; Alexander-Bloch et al., 2013). In graph theory terms, a network is a collection of nodes that are interconnected by edges. Nodes usually represent brain regions, while edges represent (structural or functional) connections. A pre-requisite to connectivity analysis is the proper designation of nodes as distinct gray matter regions. Various parcellation schemes have been proposed, including approximating Brodmann areas based on imaging-derived surrogates of myelination (Glasser and Van Essen, 2011; Bock et al., 2013), sulcation-based atlases (Van Essen, 2005; Desikan et al., 2006), high-resolution parcellations (Hagmann et al., 2008; Honey et al., 2009), as well as schemes that take the imaging voxels/vertices themselves as nodes (Lohmann et al., 2010; Tomasi and Volkow, 2011). In addition, several studies have also used data-driven techniques such as independent brain components to define network nodes (Yu et al., 2011b, 2013). Nodal definitions have shown to have a large influence on graph-theoretical parameters (Tohka et al., 2012), and the definition of reliable, biological meaningful parcellations schemes continues to be an active area of current research (Geyer et al., 2011; Glasser and Van Essen, 2011; Van Essen et al., 2012).

**Figure 3:**
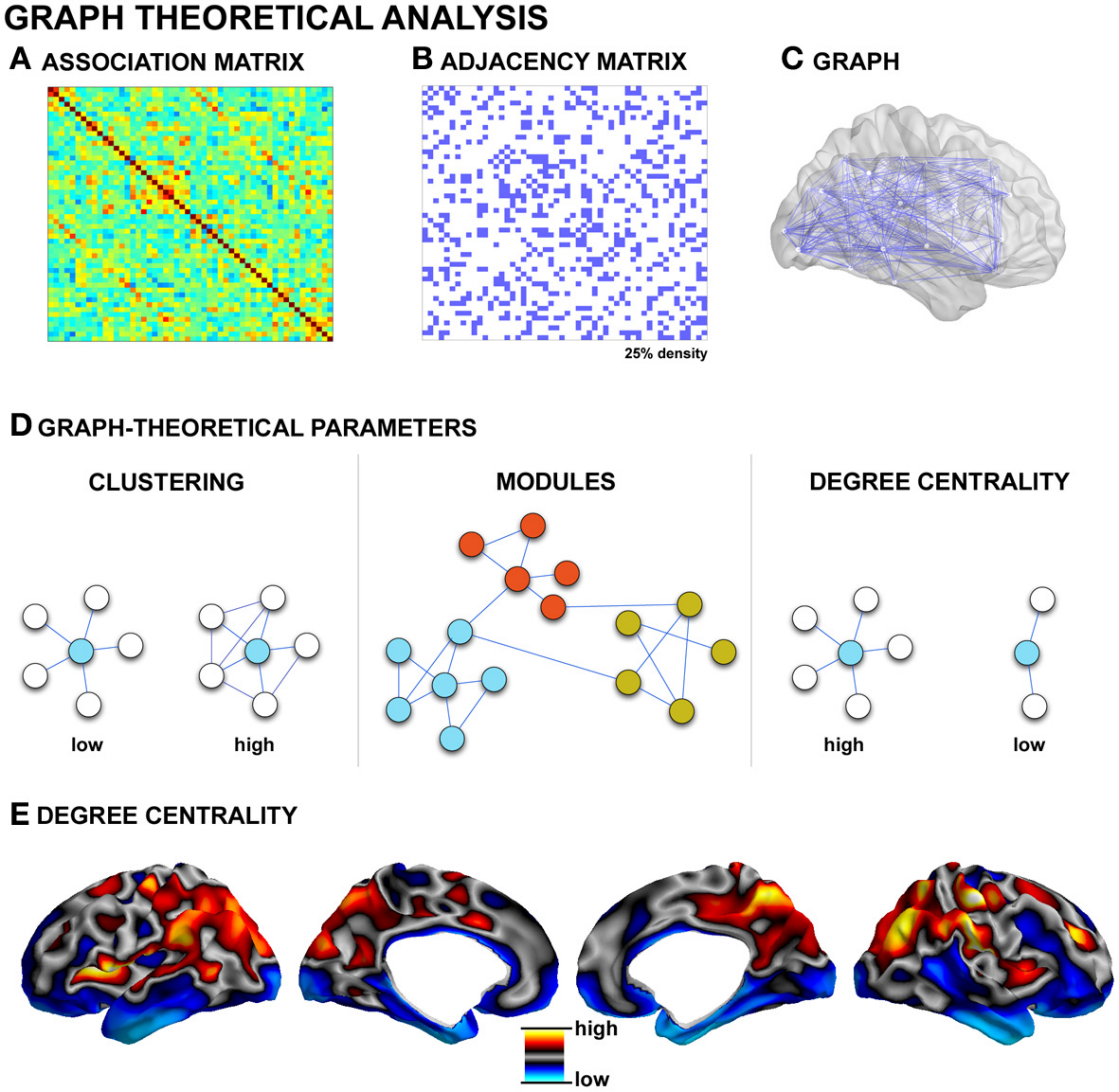
**Graph-theoretical analysis. (A)** Association matrix quantifying the degree of connectivity (derived from techniques such as diffusion MRI tractography, structural covariance, or functional connectivity, see Figure 2). **(B)** Matrices commonly undergo a thresholding procedure to remove spurious edges. In this example, the highest 25% of associations are preserved, leading to a binary adjacency matrix. **(C)** Each binary matrix is equivalent to an undirected graph. **(D)** Topological parameters such as the clustering coefficient and path length can then be measured; networks can be partitioned in modules based on groupings of connectivity among nodes; hubs can be identified, for example, as nodes with high degree centrality (i.e., a high number of connections). **(E)** Cortical degree centrality map, based on resting-state functional connectivity data from a single healthy control subject.

As described in previous sections, *in vivo* studies provide several definitions for network edges, both in the structural and functional domain (*see* Figure 3, for an example of functional and structural network generation). Accordingly, graph-theoretical analysis has been conducted across various modalities such as functional MRI (Salvador et al., 2005; He et al., 2009; Honey et al., 2009), electrophysiology (Stam, 2004; Bassett et al., 2006), diffusion-weighted MRI (Hagmann et al., 2007, 2008; Iturria-Medina et al., 2007; Gong et al., 2009), and structural covariance (He et al., 2007; Bassett et al., 2008; Chen et al., 2008; Bernhardt et al., 2011). Collectively, these studies have shown that the global topology of brain networks in healthy populations is neither random nor regular, but characteristic of a *small-world*. Small-world networks are defined by clusters of tightly inter-connected nodes, which are themselves linked to other clusters through few inter-connector links. This architecture results in overall short path lengths between individual nodes and an overall high degree of clustering (Watts and Strogatz, 1998), an architecture that enables both the specialization and integration of information transfer at relatively low wiring costs (Sporns et al., 2004). Graph-theoretical methods may be used to examine intermediary levels of organization. Communities (also called modules) are groups of nodes that are richly connected to one another within the larger framework of the entire network (Meunier et al., 2010; Bullmore and Bassett, 2011). Modularity is one of the most ubiquitous properties of complex, large-scale networks (Bullmore and Sporns, 2009), and modules may express some degree of hierarchical organization (Bassett et al., 2008; Meunier et al., 2010; Bullmore and Bassett, 2011). Theoretically, there are several advantages to a modular and hierarchical organization, including greater adaptability and robustness to changing environmental conditions (Meunier et al., 2010). Moreover, it has been suggested that the more modular and hierarchically organized a system is, the more diverse its functional activation patterns (Alexander-Bloch et al., 2010; Kaiser and Hilgetag, 2010). Modularity may be undermined by disease processes, as suggested by disrupted modularity in schizophrenia (Bassett et al., 2008; Alexander-Bloch et al., 2010; Yu et al., 2011a), and frontal lobe epilepsy (Vaessen et al., 2012a).

Besides the characterization of global and modular properties of large-scale networks, graph-theoretical techniques allow the localization of key regions within the network layout, so-called *hubs*, through centrality-based metrics (Bullmore and Sporns, 2009; Van Den Heuvel and Sporns, 2011; Zuo et al., 2012). According to formulations of centrality, hubs can be defined as regions with a high degree centrality, which means that they have a high number of connections to other nodes (Zuo et al., 2012); they can be identified on the basis of high betweeness centrality, which signifies they are located along pathways of efficient information flow (Zuo et al., 2012); finally, they can be identified through a high eigenvector centrality, which is a recursive formulation quantifying connections to other highly connected hubs (Lohmann et al., 2010; Zuo et al., 2012). Depending on their embedding in specific modules and connectivity profiles, hubs can be further classified as to whether they primarily mediate within- or between-module connectivity (Sporns et al., 2007). Assessing hubs promises to highlight critical key regions in structural and functional networks, and may thus provide a better understanding of their potential role in pathological processes (Bullmore and Sporns, 2012). In Alzheimer's disease, for example, functional hubs coincide with regions of high amyloid-beta deposition (Buckner et al., 2009). Central hubs may form a so-called *rich club*, a collection of mutually densely linked nodes with disproportionally high centrality (Van Den Heuvel and Sporns, 2011; Harriger et al., 2012). This architecture is thought to contribute to the robustness of the core constituents of the brain network. In humans, a rich club inferred from diffusion MRI has been shown to comprise lateral prefrontal, midline and lateral parietal, as well as the hippocampus, putamen, and thalamus (Van Den Heuvel and Sporns, 2011). In the macaque monkey, rich club regions have been shown to be preferentially located on short paths through the network, thereby contributing effectively to global communication (Harriger et al., 2012).

In focal epilepsy, relatively few studies have employed graph-theoretical analysis of brain networks derived from MRI (Liao et al., 2010; Bernhardt et al., 2011; Bonilha et al., 2012; Vaessen et al., 2012b). We previously showed that in drug-resistant TLE, structural networks derived from inter-regional MRI-based cortical thickness correlations are characterized by increased clustering and path length, a finding indicative of a more regular global topology (Bernhardt et al., 2011). These findings were complemented by the parallel observation of reduced network robustness, a measure of organizational stability (Bernhardt et al., 2011), and subtle alterations in the distribution of network hubs pointing toward a more paralimbic distribution in patients relative to controls. In a longitudinal study, we showed that structural network disruptions intensify over time. Furthermore, relating network parameters to postsurgical seizure outcome data indicated that patients who continued to have seizures after surgery had more marked network disruptions relative to those who became seizure-free (Bernhardt et al., 2011). These findings speak to the hypothesis that seizure recurrence after surgery may, in part, be related to an extended epileptogenic network (Ryvlin, 2003; Bernhardt et al., 2010). Our findings therefore suggest a possible clinical potential for network data in the presurgical workup.

Our finding of a more regularized topology of structural cortico-cortical networks in drug-resistant TLE closely resembled results from graph-theoretical analyses of intracerebral EEG recordings during focal seizures (Ponten et al., 2007; Kramer et al., 2008; Schindler et al., 2008) and scalp EEG data of generalized absence seizures (Ponten et al., 2009). Indeed, electrophysiological work has suggested that seizures may be associated with a sudden regularization of functional networks. It may, thus, be plausible that network synchronization during the ictal phase may influence mutual inter-cortical trophic exchanges, ultimately leading to progressive and long-lasting remodeling of inter-regional structural networks.

Few other studies assessed the topology of brain networks derived from MRI in TLE using graph-theoretical methods. A diffusion MRI tractography of structures belonging to the limbic network showed increased clustering and centrality in 12 patients with unilateral TLE (Bonilha et al., 2012). Analysis of whole-brain resting-state functional networks in patients with bilateral TLE has revealed both atypical low-level connectivity (i.e., increases in temporal and decreases in fronto-parietal connectivity) and topological disruptions, indicative of decreased clustering and path length (Liao et al., 2010). These studies have further demonstrated that TLE is associated with topological disruptions in large-scale structural and functional networks. Results have nevertheless been somewhat divergent, possibly as a result of heterogeneous patient populations and diverse network construction methods.

## Conclusions and future directions

Advances in brain network construction and graph theoretical modeling have permitted a characterization of topological aspects of healthy and abnormal brain connectivity. While an extensive literature has shown regional as well as low-level connectional disruptions in TLE, studies focusing on disruptions of network topology have been so far rather sparse. The unified and elegant framework of graph theoretical analysis promises to further consolidate our understanding of how functional networks interact with their structural substrate. Moreover, given initial observations of a relationship between the extent of network damage and post-surgical seizure outcome, establishing subject-specific profiles of network properties has great potential to assist in clinical decision-making.

### Conflict of interest statement

The authors declare that the research was conducted in the absence of any commercial or financial relationships that could be construed as a potential conflict of interest.
